# Is retrospective assessment of health-related quality of life valid?

**DOI:** 10.1186/s12891-020-03434-8

**Published:** 2020-06-30

**Authors:** Andrew Lawson, Aidan C. Tan, Justine Naylor, Ian A. Harris

**Affiliations:** Whitlam Orthopaedic Research Centre, Level 2, Ingham Institute for Applied Medical Research South Western Sydney Clinical School, UNSW Sydney, 1 Campbell St, Liverpool, Sydney, NSW AUS 2170 Australia

**Keywords:** Quality of life, EQ-5D, Test-retest reliability, Validity, Reproducibility of results, Prospective, Retrospective, Surveys and questionnaires

## Abstract

**Background:**

Health-related quality of life (HRQoL) is a commonly used health outcome. For many acute conditions (e.g. fractures), retrospective measurement of HRQoL is necessary to establish pre-morbid health status. However, the validity of retrospective measurement of HRQoL following an intervening significant health event has not been established. The aim of this study was to test the validity of retrospective measurement (recall) of HRQoL by using a test-retest design to measure reliability and agreement between prospective and retrospective patient-reported HRQoL before and after an intervening health event (elective orthopaedic surgery).

**Method:**

Participants were recruited from the pre-admission clinic of a metropolitan hospital. Participants were assessed for their HRQoL using the EQ-5D-5L at two time-points; prospectively at 2 weeks prior to their date of surgery and then retrospectively (recalling their pre-operative health) following elective hip or knee joint replacement surgery. Prospective measurements were compared with retrospective measurements for the five domain scores (nominal data) using intra-class correlation and for the EQ-Index score and EQ-Visual Analogue Scale (VAS) score (continuous data), using Pearson’s correlation. Agreement was tested in continuous variables using Lin’s coefficient of concordance (p_c_) and Bland-Altman plots.

**Results:**

One hundred seventy-four patients consented to participate. Eighty-eight paired prospective and retrospective scores were collected and there was a median between-test period of 15 days. At a group level, the prospective measurements were similar to the retrospective measurements; the modes and means of the five domain scores were not different and the mean differences (MD) between the scores for EQ-Index (MD = 0.02, on a scale of 0–1) and EQ-VAS (MD = 0.53, on a scale of 1–100) were negligible. However, the correlation of paired scores was varied; the range of domain score correlations was 0.52 to 0.74, the concordance was substantial for the EQ-Index scores (p_c_ = 0.76, 95% CI = 0.66, 0.84) and moderate for the EQ-VAS scores (p_c_ = 0.46, 95% CI = 0.28, 0.61).

**Conclusion:**

Agreement between prospective and retrospective measurements was high at a group level and moderate to substantial at an individual level. Retrospective measurement of HRQoL using the EQ-5D-5L in an orthopaedic clinical context is a valid alternative to using reference data to estimate baseline or pre-morbid health status.

## Background

Health related quality of life (HRQoL) is an assessment and expression of subjective well-being that includes emotional, social and physical aspects. HRQoL can be used for health economic purposes and as an outcome measure in the treatment of health conditions. HRQoL is measured at an individual level; it is not directly observable and is only deducible from patients’ responses to questionnaires [1].

The EuroQol instruments are non-disease specific examples of multi-dimensional tools to measure HRQoL and the most widely applied version is the EQ-5D [2] with the five-level version (EQ-5D-5L) the most recent version. The EQ-5D-5L produces both categorical (five dimensions) and continuous data (utility index score and visual analogue scale, VAS, score). The five descriptive dimensions are combined according to a scoring algorithm to produce a quantitative index score (EQ-index) that can be compared to normative values. The index scores range from 1 (full health) to 0 (equivalent to death), with scores less than 0 defined as health states worse than death. Population norms for EQ-index scores have been reported for various populations around the world but none are available for Australia [2]. The EQ-5D VAS can be used as an outcome in itself or to help weight the index scores.

The EQ-5D is commonly used and has been widely compared with other instruments and validated in musculoskeletal health-specific contexts. A recent systematic review demonstrated good reliability and validity and moderate responsiveness [3]. Two studies using cohorts of patients being treated for carpal tunnel syndrome found excellent reliability [4], strong validity and minimal bias for age and gender [5]. The properties of the EQ-5D have also been tested in musculoskeletal health-specific contexts such as proximal humeral fractures [6], adolescent idiopathic scoliosis [7], rheumatoid arthritis [8–10], lower back surgery [11] and knee osteoarthritis [12, 13] with similar outcomes reported.

The EQ-5D is only recommended for prospective use. The EuroQol instruments are framed in the present tense and there are no retrospective versions available. In a clinical context, unless the health condition is foreseeable, the clinician or health researcher does not have a baseline measure of the patient’s pre-morbid health state. Normative population-level, non-disease specific data can be used, or retrospective measurement of baseline health status can be measured. However, this is susceptible to recall bias. In a health economics context, the retrospective assessment of health status, gathered for the purpose of estimating healthy life expectancy has been shown to closely approximate estimates based on prospective health information [14]. Numerous studies have investigated the test-retest reliability of the EQ-5D [15–20] and other studies have supported the use of retrospective measurement of baseline data following a health event or an intervention [21–23], and retrospective measurement in preference to using population norms in determining baseline health status [24, 25]. However, the validity of the retrospective use of the EQ-5D has not been established.

The primary aim of this study was to use a significant health event, elective hip or knee joint replacement surgery, to determine the validity of the retrospective measurement of self-reported health status using the EQ-5D-5L, comparing it to prospective measurement.

## Methods

### Recruitment and data collection

This study was designed as a test-retest reliability study with the test being the prospective (contemporaneous) measure of pre-operative health and the retest being the retrospective (recalled) measure of pre-operative health using EQ-5D, recorded after a major intervening health event. The tests were conducted 2 weeks apart and with elective hip or knee joint replacement surgery occurring in between. The study was conducted at The Sutherland Hospital, Sydney, Australia. Ethics approval was granted by the hospital ethics committee (LNR/17/POWH/384) and permission was granted by EuroQoL for the use of the EQ-5D-5L in this study.

Patients were screened through the elective surgery waitlist and recruited in-person from the pre-admission clinic. Patients were eligible for inclusion if they were (a) aged 18 years and older, and (b) presented for an elective hip or knee arthroplasty (e.g. total hip replacement or, knee replacement including bilateral and unicompartmental knee replacement). Patients were excluded if they were (a) unable to consent because they were cognitively impaired or not proficient in English, (b) unable to be contacted by telephone because they were hearing impaired or did not own a phone, or (c) unsuitable to be assessed prospectively because their planned date of operation was within 10 days of presentation or they planned on being overseas during the prospective period. At recruitment, participants were shown the format of the EQ-5D-5L and were given a hardcopy as part of the patient information consent form, so that they were familiar with the question format for telephone interview due to occur 2 weeks prior to planned surgery.

Participant data were collected relating to identifying information (name, date of birth, sex and medical record number), contact details (primary and secondary phone numbers), eligibility status, participation status (consented, declined or missed), primary language, operation type, important dates (pre-admission clinic, planned operation and actual operation), and the prospective and retrospective EQ-5D-5L (date of administration and scores). The prospective measurement was conducted by telephone 2 weeks pre-operatively by an investigator (AT). Participants completed the unmodified EQ-5D-5L by telephone with respect to their current, pre-operative health status.

The retrospective measure was administered verbally, in-person at the hospital post-operatively (AL, AP and MK), prior to their discharge from hospital. Participants were asked to recall their pre-treatment health status as it was 2 weeks prior to their surgery using a modified EQ-5D-5L (past tense) questionnaire administered verbally.

### Statistical analysis

A sample size of 77 was determined using Zou’s (2012) sample size calculation incorporating assurance probability [26]. A sample size of 77 ensured a 90% assurance probability given the half width of a 95% two-sided confidence interval for a correlation or concordance of 0.8.

The retrospective EQ-5D-5L measurements were compared with prospective measurements; the EQ-5D-5L produces seven separate outcomes (5 domain scores, an index score and a VAS score) and each prospective outcome was paired with its retrospective outcome and tested for correlation. The five domain scores were assessed for intra-class correlation (ICC). The domain scores were then converted to an index score using a scoring algorithm estimated from a sample of the United Kingdom (UK) adult general population, using an EQ-5D-5L calculator. The calculator was developed by Sheffield Hallam University on behalf of The Chartered Society of Physiotherapy (United Kingdom) in 2011 [27]. The purpose of the tool is to enable illustration of change in quality of life as a result of physiotherapy interventions. However, in this instance, we used the tool to pair prospective and retrospective HRQoL scores and to display the distribution of difference in domains scores. The prospective index scores and VAS scores were correlated with the retrospective scores. Agreement was assessed for the continuous variables (EQ-index and EQ-VAS scores). Bland-Altman plots were made with 95% limits of agreement (LOA) and Lin’s concordance correlation coefficient (p_c_) was calculated. To interpret the results for correlation and for agreement, we used benchmarks defined by Cicchetti (1994), whereby 0.21 to 0.4 represented ‘fair’, 0.41–0.60 was ‘moderate’, 0.61–0.80 was ‘substantial’ and 0.81–1.00 was ‘almost perfect’ reliability [28].

## Results

### Participant characteristics

Three hundred fifty-five patients attended the pre-admission clinic for joint replacement surgery at our facility in the period from 27 September 2017 to 28 September 2018 and 291 patients were screened for eligibility, of which 78 were ineligible, 39 declined to participate and 174 consented to participate. Prospective outcomes were gathered on 144 participants and retrospective outcomes were gathered on 104 participants. Both prospective and retrospective outcomes were gathered for 88 participants. The median time between tests was 15 days, the range was 3 to 64 days and the mean follow-up was 19 days.

**Fig. 1:**
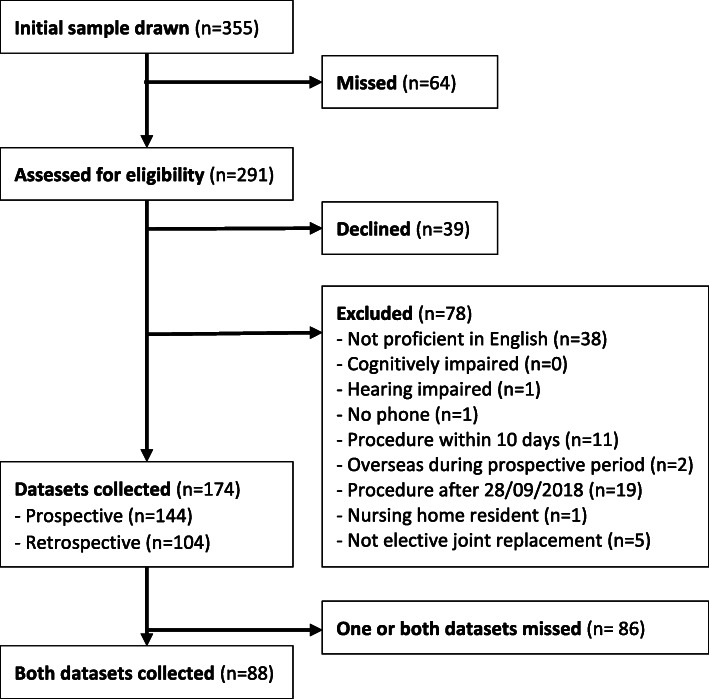
Participant flow diagram

The main reason for missing prospective measurements on recruited participants at the pre-operative stage was that the participant could not be contacted by their primary or secondary telephone number after five attempts on consecutive weekdays. The main reason for missing retrospective measurements on post-operative participants was that the patient’s date of surgery was changed or cancelled after their attendance at pre-admission clinic. Other reasons included that the patient required an escalation in their medical treatment after surgery (e.g. critical care admission) or they were discharged from the hospital before their follow-up. Participant flow is shown in Fig. 1.

The demographic characteristics of the study sample are described in Table 1. The mean age of participants was similar to non-participants. There were slightly more female participants (51%) than male participants and there were more knee replacements (67%) than hip replacements (33%) in this sample. English was the predominant first language for both participants and non-participants but, given that English-proficiency was an inclusion criterion for participation, the proportion of first languages other than English was higher in the population (26%) than in the study sample (8%).

**Table 1:** Participant characteristics

**Sample** (*n* = 364)		**Participants** (*n* = 88)	**Non-participants** (*n* = 276)
**Age (years)**	Mean (range)	68 (32–88)	68 (26–91)
**Sex**	Male	43 (49%)	(unknown)
	Female	45 (51%)	(unknown)
**First Language**	English	81 (92%)	203 (74%)
	Other than English	7 (8%)	73 (26%)
**Procedure**	Hip replacement	29 (33%)	(unknown)
	Knee replacement	59 (67%)	(unknown)
**Dates**	Median (range) duration between prospective and retrospective dates (days)	15 (3 to 64)	

### Categorical variables

Comparisons of the nominal data are displayed in Table 2. The medians and the modes for each of the five dimensions remained unchanged between the prospective and the retrospective measurements, indicating negligible difference between prospective and retrospective measurements at a group level.

**Table 2:** Descriptive statistics for prospective and retrospective measures

**Variable**	**Domain**					**EQ-5D-5L Index**	**EQ-VAS**
	**Mobility**	**Self-Care**	**Usual Activities**	**Pain/ Discomfort**	**Anxiety/ Depression**		
**Prospective** (*n* = 88)							
**Mean**						0.4691	66.89
**Median(range)**	3.0 (1 to 4)	2.0 (1 to 4)	3.0 (1 to 5)	3.0 (1 to 5)	2.0 (1 to 5)	0.5135 (−0.1994 to 1)	70 (10 to 100)
**Mode**	3	1	3	3	1		
**Retrospective** (*n* = 88)							
**Mean**						0.4470	66.36
**Median (range)**	3.0 (1 to 5)	2.0 (1 to 4)	3.0 (1 to 5)	3.0 (2 to 5)	2.0 (1 to 5)	0.5197 (−0.1464 to 0.8366)	70 (5 to 95)
**Mode**	3	1	3	3	1		

The distribution of difference from prospective to retrospective measurements in each of the five dimensions is illustrated in Appendix 1. The distribution of change provides an indication of the magnitude and direction of difference in scores in each dimension from prospective to retrospective measurements. The range of agreement in paired prospective and retrospective scores was 48% (usual activities) to 65% (mobility).

The results gathered from each of the five EQ dimensions are presented in 5 × 5 tables (Appendix 2). Intraclass correlation coefficient (ICC) scores were reported for each of the five domains. Correlation was moderate for usual activities (*r* = 0.52) and anxiety/depression (*r* = 0.54) and was substantial for mobility (*r* = 0.74), personal care (*r* = 0.62) and pain/discomfort (*r* = 0.65).

### Continuous variables

**Fig. 2:**
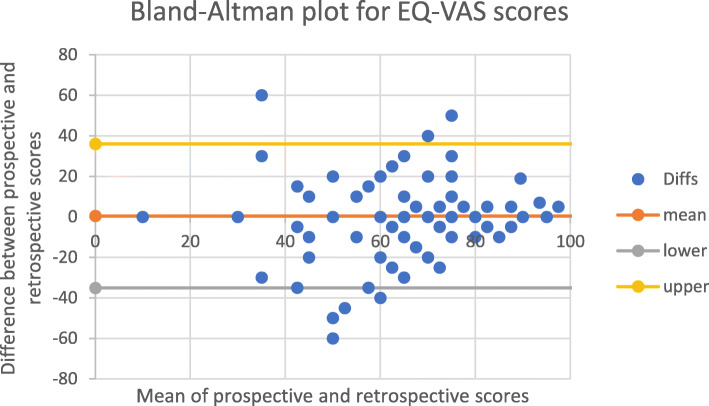
Bland-Altman plot for EQ-VAS scores

**Fig. 3:**
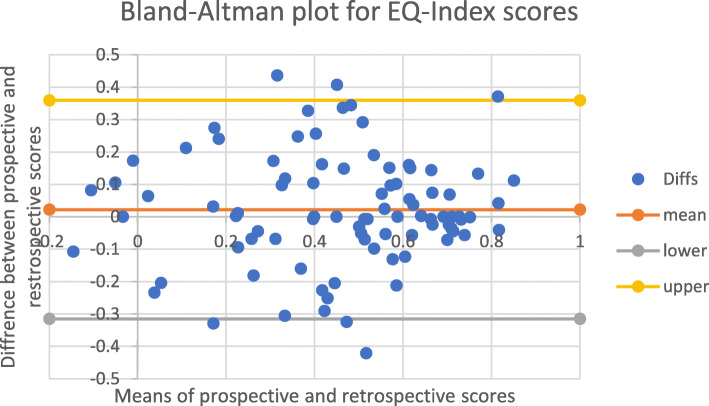
Bland-Altman plot for EQ-Index scores

The mean differences (MDs) for each of the continuous variables were negligible; 0.53 on a scale of 0 to 100 for the EQ-VAS and 0.02 on a scale of 0 to 1 for the EQ-Index. This indicated strong agreement between prospective and retrospective measures at a group level. However, the Bland-Altman plots and Lin’s concordance (p_c_) for paired EQ-VAS and EQ-Index scores indicated that agreement at an individual level was lower. The Bland-Altman plots for EQ-VAS (Fig. 2) and EQ-Index (Fig. 3) scores show that the 95% LOA. around the MD were − 32.34 to 33.38, on a scale of 0–100 for the EQ-VAS and − 0.31 to 0.36 on a scale of 0–1 for the EQ-Index. Agreement was moderate for EQ-VAS (p_c_ = 0.46 95% CI, 0.28, 0.61) and substantial for EQ-Index scores (p_c_ = 0.76 95% CI, 0.66, 0.84) (Table 3).

**Table 3:** Correlation and agreement for continuous variables

*n* = 88	**EQ-VAS**	**EQ-index**
**Mean difference** (LOA)	0.53 (−32.34, 33.38)	0.02 (− 0.31, 0.36)
**Pearson CC** (r [p])	0.46 (*p* < 0.0001)	0.76 (*p* < 0.0001)
**Lin’s CCC** (p_c_ [95% CI])	0.46 (0.28, 0.61)	0.76 (0.66, 0.84)

## Discussion

The results of this study showed that retrospective measurement of HRQoL produced equivalent results to prospective measurement at a group level but agreement was lower at an individual level. We could find no directly comparable studies but numerous recent studies investigating test-retest reliability of the EQ-5D-5L tool (not using recall of health status) have reported similar differences between the two testing periods (Table 4). A 2018 study surveyed a cross-section of the Indonesian general adult population [15] with a mean between-test period of 17 days. The findings were similar to our findings in that the group-level correlations were high for the five dimensions (ICC = 0.85–0.99) but that agreement for the EQ-VAS and EQ-Index scores was much lower (p_c_ = 0.45 and 0.37 respectively). An English study examined the test-retest reliability of the EQ-5D-3L in the UK general population and reported an ICC of 0.83 in the EQ-Index scores [16]. A Chinese study of carers of cancer patients reported high test-retest reliability for EQ-VAS and EQ-Index scores of 0.87 and 0.99 respectively [17]. However, the follow-up between test and retest in that particular study was only 1 day meaning the results were likely to be heavily influenced by recall bias compared with the results of our study. Other studies with similar follow-up periods to our study have reported test-retest ICCs for EQ-Index scores of 0.78 [18], 0.70 [19] and 0.75 [20]. These results would lead us to suggest that retrospective measurement of baseline health status produces similar test-retest reliability compared to studies measuring current (prospective) health status at a similar interval.

**Table 4:** studies of test-retest reliability of EQ-5D

Study	Language	Population	Follow-up	Reliability
Current study	English	Elective orthopaedic surgery patients	Mean of 19 days	CCC of 0.46 for EQ-VAS CCC of 0.76 for EQ-Index
Purba, 2018 [15]	Indonesian	General population	Mean of 17 days	ICC and CCC of 0.45 for EQ-VAS ICC and CCC of 0.37 for EQ-Index
Al-Janabi, 2015 [16]	English	UK general ppulation	14 days	ICC of 0.83 for EQ-Index
Li, 2019 [17]	Chinese	Carers of cancer patients	24 h	ICC of 0.99 for EQ-Index ICC of 0.87 for EQ-VAS
Cheung, 2016 [18]	Chinese	Scoliosis patients	Mean of 20 days	ICC of 0.78 for EQ-index score
Pattanaphesaj, 2015 [19]	Thai	Diabetes patients	Approx. 3 weeks	ICC of 0.70 for EQ-Index
Kim, 2013 [20]	Korean	General population	Mean of 19 days	ICC of 0.75 for EQ-index score

Similar findings have been reported in the context of testing the validity of different modes of administration of patient-reported measures. In a 2017 study, the equivalence of patient-completed and telephone interview modes of administration of the EQ-5D-5L were tested in a cohort of orthopaedic patients [29]. The equivalence was established according to the minimum important difference for the index and VAS scales but correlation between paired domain scores were similar to or lower than those reported in our study. Another study from 2014 tested the correlation of mail and telephone administration of the Oxford hip and knee scores in an orthopaedic cohort [30], showing that the two different modes of administration produced equivalent results at a group level but that agreement was low at an individual level.

### Bias

Both prospective and retrospective measurements of difference in self-reported health status are susceptible to bias. Prospective measurements are subject to scale recalibration, a changed conceptualisation of the answer scale secondary to changed internal standards of construct interpretation and judgement from pre-test to post-test [1]. Significant intervening health events can exaggerate this bias by shifting the frame of reference and catalysing a revaluation of prior and present health status [31]. In the case of this study, the retrospective measurement might have been biased by factors such as general anaesthesia, the trauma of surgery or post-operative levels of pain. Schwartz & Sprangers (1999) suggest that this scale recalibration makes the retrospective measurement of change in self-reported health status more appropriate than the prospective measurement of change in self-reported health status because both recalled and current health status are evaluated using a consistent internal standard of construct interpretation and judgment [32].

Alternatively, retrospective measurement may be subject to recall bias, the incorrect self-assessment of former health status due to inaccurate or incomplete recollection [1]. Recall bias occurs because recollection is a reconstructive and inferential process that is subject to errors, losses, distortions and psychological processes in the present state and over time [1]. Bias results in underestimations or overestimations of former health status which may be non-directional, occurring by chance and cancelling out on average, or directional, consistently occurring and producing a unidirectional error. In this study, recall might have been biased by general anaesthesia, the trauma of surgery or the side effects of analgesia. To reduce the chance of recall bias, retrospective measures were taken as close to the patients discharge from hospital as possible. Given that our results were very similar to other test-retest studies that didn’t involve an intervening health event and that had similar time between test and retest, it is unlikely that our results were affected significantly by scale recalibration or recall bias. That said, though not the aim of the study, the use of a control group that was not exposed to an intervening health event would have provided evidence either way about the presence of such bias.

### Strengths and limitations

The aim of this study was to investigate the validity of the retrospective use of HRQoL. Many validation studies test for reliability using correlation but do not necessarily test for agreement using concordance and this can be problematic in the context of longitudinal studies [33]. Correlation measures the strength of the linear relationship between two measures but does not measure the equality between paired sets of values. This study used Bland-Altman plots and assessment of concordance to investigate agreement between prospective and retrospective measures.

The main limitation of this study was the low follow-up rate of participants. The investigators made efforts to maximise the follow-up rate by excluding patients who were less likely to be contactable by phone and by tracking patient journeys in the electronic medical record. The first EQ-5D-5L questionnaire was administered via telephone and the second EQ-5D-5L questionnaire was administered face-to-face which increased the risk of detection bias. The risk of bias was minimised by investigators using standardised explanations and delivering both surveys verbally. Another limitation was the lack of a control group which meant that the risk of scale recalibration or recall bias could not be assessed.

## Conclusions

The validity of measuring HRQoL retrospectively has not previously been assessed. Our results indicate that retrospective measurement of HRQoL, using the EQ-5D-5L in an elective orthopaedic clinical context provides results that are almost equivalent to prospective measurement at a group-level but not at an individual level. These results are similar to the results of studies investigating test-retest reliability of EQ-5D-5L, suggesting that the retrospective measurement of HRQoL to estimate pre-morbid health status is valid.

## Figures and Tables

**1.1:**
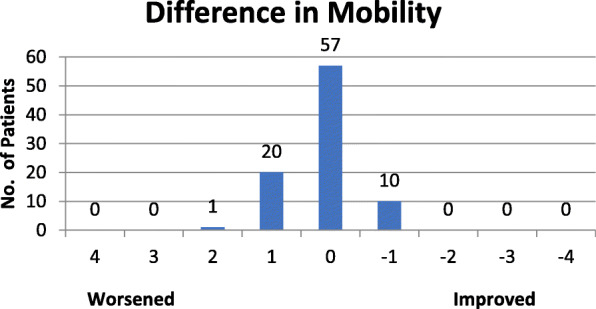
Distribution of difference in EQ-mobility scores

**1.2:**
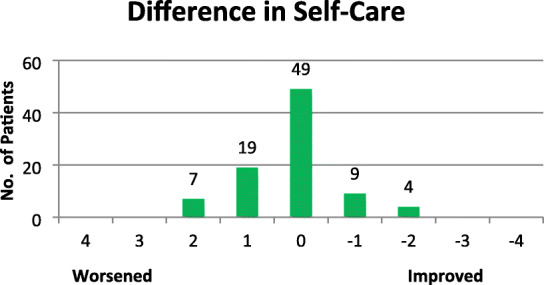
Distribution of difference in EQ-self-care scores

**1.3:**
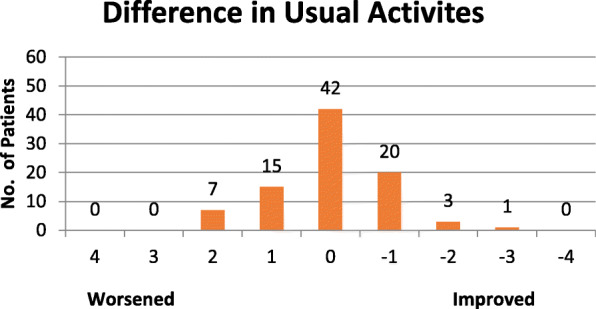
Distribution of difference in EQ-usual activities scores

**1.4:**
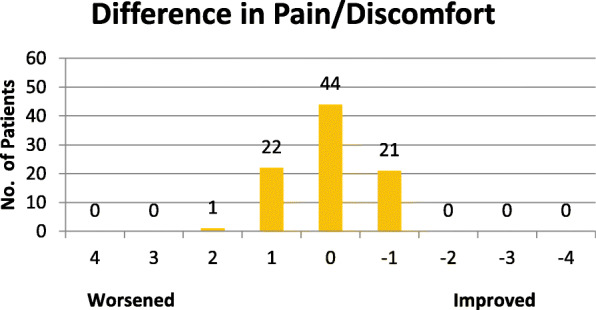
Distribution of difference in EQ-pain/discomfort scores

**1.5:**
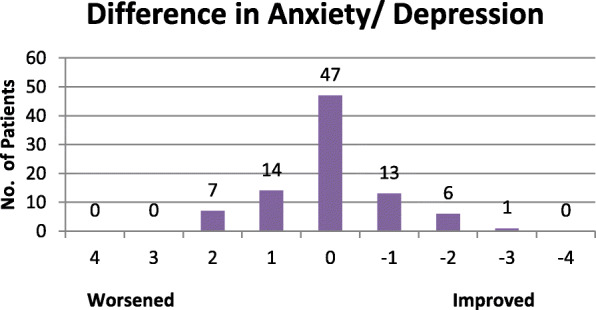
Distribution of difference in EQ-anxiety/depression scores

## Data Availability

The datasets used and/or analysed during the current study are available from the corresponding author on reasonable request.

## Appendix 2

**2.1:** Reliability of Mobility question

		Retrospective					
		1	2	3	4	5	
**Prospective**	1	3	0	1	0	0	4
	2	1	9	8	0	0	18
	3	0	3	24	11	0	38
	4	0	0	6	21	1	28
	5	0	0	0	0	0	0
		4	12	39	32	1	88
ICC = 0.74							

**2.2:** Reliability of Personal Care question

		Retrospective					Totals
		1	2	3	4	5	
**Prospective**	1	27	10	5	0	0	42
	2	2	8	6	2	0	18
	3	3	5	11	3	0	22
	4	0	1	2	3	0	6
	5	0	0	0	0	0	0
**Totals**		32	24	24	8	0	88
ICC = 0.62							

**2.3:** Reliability of Usual Activities question

		Retrospective					Totals
		1	2	3	4	5	
**Prospective**	1	4	2	3	0	0	9
	2	1	8	5	2	0	16
	3	3	9	22	6	2	42
	4	1	0	7	7	2	17
	5	0	0	0	3	1	4
**Totals**		9	19	37	18	5	88
ICC = 0.52							

**2.4:** Reliability of Pain/Discomfort question

		Retrospective					Totals
		1	2	3	4	5	
**Prospective**	1	0	3	1	0	0	4
	2	0	5	6	0	0	11
	3	0	8	18	12	0	38
	4	0	0	11	17	1	29
	5	0	0	0	2	4	6
Totals		0	16	36	31	5	88
ICC = 0.65							

**2.5:** Reliability of Anxiety/Depression question

		Retrospective					Totals
		1	2	3	4	5	
**Prospective**	1	29	7	5	0	0	41
	2	3	9	4	1	0	17
	3	4	8	7	3	1	23
	4	1	0	2	2	0	5
	5	0	0	2	0	0	2
**Totals**		37	24	20	6	1	88
ICC = 0.54							

## Abbreviations

CI: Confidence interval
EQ-5D-5L: EuroQol 5-dimension 5-level
EQ-Index: EuroQol-5D health utilities Index
EQ-VAS: EuroQol-Visual Analogue Scale
HRQoL: Health-related quality of life
ICC: Intra-class correlation
LOA: Limits of agreement
MD: Mean difference
p_c_: Lin’s coefficient of concordance
UK: United Kingdom
